# Calcium Promotes the Formation of Syntaxin 1 Mesoscale Domains through Phosphatidylinositol 4,5-Bisphosphate*

**DOI:** 10.1074/jbc.M116.716225

**Published:** 2016-02-16

**Authors:** Dragomir Milovanovic, Mitja Platen, Meike Junius, Ulf Diederichsen, Iwan A. T. Schaap, Alf Honigmann, Reinhard Jahn, Geert van den Bogaart

**Affiliations:** From the ‡ Department of Neurobiology, Max Planck Institute for Biophysical Chemistry, 37077 Göttingen, Germany,; the § Department of Neuroscience, Program in Cellular Neuroscience, Neurodegeneration, and Repair, Howard Hughes Medical Institute, Yale University School of Medicine, New Haven, Connecticut 06511,; the ¶ Third Institute of Physics, Faculty of Physics,; ‖ Institute for Organic and Biomolecular Chemistry, Georg August University, 37077 Göttingen, Germany,; the ** School of Engineering and Physical Sciences, Heriot-Watt University, Edinburgh EH14 4AS, United Kingdom,; the ‡‡ Max Planck Institute for Molecular Cell Biology and Genetics, 01307 Dresden, Germany, and; the §§ Department of Tumor Immunology, Radboud University Medical Center, 6525 GA Nijmegen, The Netherlands

**Keywords:** calcium, membrane structure, plasma membrane, protein-lipid interaction, SNARE proteins, PI(4,5)P_2_, clustering, membrane domains, syntaxin 1

## Abstract

Phosphatidylinositol 4,5-bisphosphate (PI(4,5)P_2_) is a minor component of total plasma membrane lipids, but it has a substantial role in the regulation of many cellular functions, including exo- and endocytosis. Recently, it was shown that PI(4,5)P_2_ and syntaxin 1, a SNARE protein that catalyzes regulated exocytosis, form domains in the plasma membrane that constitute recognition sites for vesicle docking. Also, calcium was shown to promote syntaxin 1 clustering in the plasma membrane, but the molecular mechanism was unknown. Here, using a combination of superresolution stimulated emission depletion microscopy, FRET, and atomic force microscopy, we show that Ca^2+^ acts as a charge bridge that specifically and reversibly connects multiple syntaxin 1/PI(4,5)P_2_ complexes into larger mesoscale domains. This transient reorganization of the plasma membrane by physiological Ca^2+^ concentrations is likely to be important for Ca^2+^-regulated secretion.

## Introduction

Neurotransmitter release requires tight spatial and temporal control. Temporal control is achieved by the interplay between Ca^2+^ influx and synaptotagmin 1, the main Ca^2+^ sensor on the synaptic vesicle (1, 2). Spatial control is achieved by the specific lateral organization of the presynaptic SNARE proteins syntaxin 1 and SNAP25 in the plasma membrane (3–5). Syntaxin 1 and SNAP25 complex with synaptobrevin 2 (VAMP2) in the synaptic vesicle, resulting in membrane fusion and the release of neurotransmitters (5, 6). It is well established that syntaxin 1 and SNAP25 are not randomly distributed over the plasma membrane of neurons and neuroendocrine cells but form clusters of ∼40–100 nm in diameter (7–9). These clusters are necessary for the recruitment of the neurotransmitter-containing vesicles to the plasma membrane (10–12). Clustering of SNAREs has been intensively studied, and different mechanisms affect their lateral organization, including both protein-protein and protein-lipid interactions (3, 4, 8, 12–14). Polyphosphoinositides belong to the components shown to be important for syntaxin domain formation. Syntaxin 1 contains a polybasic stretch juxtaposed to its transmembrane domain, which interacts electrostatically with polyphosphoinositides (15–17), including PI(4,5)P_2_,^3^ which represents more than 80% of total lipids in syntaxin 1 domains (16).

Apart from triggering synaptic vesicle release, calcium ions induce a reorganization of the plasma membrane, resulting in larger, mesoscale domains of SNAREs, including syntaxin 1 (18). This clustering relates to the net charge of the protein, with more anionic proteins forming more pronounced clusters with Ca^2+^, arguing for a charge bridging effect. In addition, it is well established that Ca^2+^, but not Mg^2+^, also induces domain formation of PI(4,5)P_2_ by means of charge bridging, which results in connection between multiple PI(4,5)P_2_ molecules (19–23). Because the polybasic juxtamembrane stretch of syntaxin 1 interacts with the multiple negative charges in the headgroup of PI(4,5)P_2_ (15–17), we hypothesized that Ca^2+^ may cluster syntaxin 1 indirectly via PI(4,5)P_2_. This would result in coalescence of multiple smaller syntaxin 1/PI(4,5)P_2_ clusters into a larger domain. Indeed, using a combination of superresolution stimulated emission depletion (STED) nanoscopy, FRET, and atomic force microscopy (AFM), we now show that, under physiological conditions, Ca^2+^ ions can act as a charge bridge and induce the formation of syntaxin 1/PI(4,5)P_2_ domains at the mesoscale.

## Experimental Procedures

### 

#### 

##### Materials

Syntaxin 1 TMD (residues 266–288; sx-1 TMD *Rattus norvegicus* sequence) and syntaxin 1 TMD mutant (sx-1 TMD with the following mutations: K265A and K266A) were synthesized using Fmoc solid-phase synthesis as described in Ref. 16. The fluorescent dyes Atto647N NHS-ester (Atto-Tec) and Rhodamine red succinimidyl ester (Life Technologies) were coupled to the N termini of the peptides during the Fmoc synthesis.

DOPC (1,2-dioleoyl-*sn-*glycero-3-phosphatidylcholine), 1,2-dioleoyl-*sn-*glycero-3-phosphatidylserine, and PI(4,5)P_2_ (1,2-dioleoyl-*sn-*glycero-3-phosphatidyl-(1′-myo-inositol-4′,5′- bisphosphate)) were purchased from Avanti Polar Lipids. Atto647N labeled at the SN1 position of PI(4,5)P_2_ and Atto590 coupled to ceramide were gifts from Dr. Vladimir Belov (MPI-BPC, Göttingen, Germany).

##### Cell Analyses

We used the pheochromocytoma cell line PC12 from *R. norvegicus* (24) to prepare native membrane sheets by gentle sonication as described in Ref. 12. Sonication buffer contained 20 mm potassium HEPES (pH 7.4), 120 mm potassium gluconate, 20 mm potassium acetate, 2 mm ATP, and 0.5 mm DTT. The antibody used for immunohistochemistry was anti-syntaxin 1 mouse IgG1 (Sigma, clone HPC-1) labeled with KK114-maleimide (a gift from Dr. Vladimir Belov, Max Planck Institute for Biophysical Chemistry).

##### FRET Measurements

For FRET measurements, we prepared large unilamellar vesicles (LUVs) that contained Rhodamine Red coupled to sx-1 TMD (donor fluorophore) and Atto647N coupled to sx-1 TMD (acceptor) as described in Ref. 25. The total protein-to-lipid molar ratio in our FRET measurements was 1:1000. Excitation was at 560 nm, and emission was collected from 570–700 nm, with 1-nm slit widths on a FluoroMax-2 fluorescence spectrometer (Horiba). We corrected for cross-talk resulting from acceptor excitation using samples containing only the acceptor fluorophore. The FRET efficiency was calculated as the ratio of emission intensities at 660 nm (acceptor maximum) over 580 nm (donor maximum).

##### STED Nanoscopy

For STED nanoscopy, a home-built setup was used, with pulsed excitation lasers at 595 and 640 nm. The fluorescence was collected from 600–640 and 660–720 nm with avalanche photo diodes (Excelitas and Micro Photon Devices). Superresolution was achieved using a STED laser (775 nm, 20 MHz pulsed fiber laser, IPG Photonics). By combining a 2π vortex phase plate (RPC Photonics) and a λ/4 plate, the typical “donut-shaped” focal intensity distribution of the STED beam was produced. Using the same STED beam for both dyes inherently ensured a colocalization accuracy far below the resolution limit (26). Another setup employed was a commercially available two-color STED setup (Abberior Instruments, Göttingen, Germany). This setup had two pulsed excitation lasers at 594 and 640 nm and a pulsed STED laser at 775 nm. The setup had a QUAD beam scanner (Abberior Instruments). Pulse energies ranging from 3–8 nJ in the back aperture of the objective yielded a lateral resolution of down to 30 nm. Data acquisition was done using ImSpector software. The density of clusters was analyzed using the particle analysis plugin in the Fiji software, and the cluster correlation was obtained using Pearson's correlation analysis from the Fiji software tools (27). Cluster sizes were calculated as the full width at half-maximum intensities (FWHM).

##### AFM Imaging of Stacked Lipid Bilayers

Glass coverslips were cleaned using a Plasma cleaner Fempto timer with a 40-kHz, 100-W generator (Diener Electronic), and a lipid/sx-1 TMD bilayer was generated by spin-coating as described in Ref. 25. The reconstituted bilayers were imaged with a Cervantes full mode AFM system (Nanotec) using AC40TS cantilevers (*f*_0_ = 110 kHz, k = 0.1 N/m, Olympus) as described previously (28). Calibration of the cantilevers was accomplished by using the thermal noise spectrum. We employed the Jumping Mode Plus (jump-off, 100 nm; sample points, 50), which allows scanning at controlled vertical forces between 0.2 nN and several nanonewtons (29).

## Results

**FIGURE 1.**
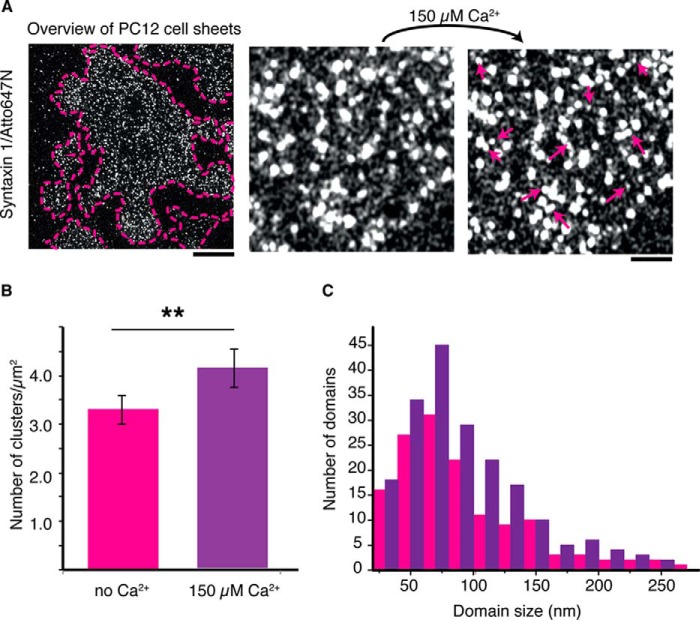
**Calcium promotes clustering of syntaxin 1 in the plasma membrane of PC12 cells.** *A*, overview of a typical PC12 membrane sheet immunostained for syntaxin 1 and imaged by STED (*left panel*, *scale bar* = 4 μm) and sections of the sheet at larger magnification both before (*center panel*) and after (*right panel*, *scale bar* = 1 μm) addition of 150 μm Ca^2+^. Newly appeared syntaxin domains after addition of Ca^2+^ are indicated by *arrows. B*, the density of syntaxin 1 clusters increased by ∼25% after addition of Ca^2+^. *Error bars* indicate the range from three independent experiments with at least 10 sheets analyzed. **, *p* < 0.01, two-sided paired *t* test). *C*, the size distributions (FWHM) of syntaxin 1 domains in the plasma membrane in the absence (*pink*) and presence (*purple*) of 150 μm Ca^2+^. Data are pooled from three independent experiments.

We first confirmed the previously reported finding (18) that elevated Ca^2+^ promotes clustering of syntaxin 1 in the plasma membrane. We employed PC12 cell sheets, which are a widely used model system for studying the lateral organization of membranes (8, 9, 12, 13, 16, 25). Using STED nanoscopy, we obtained high-resolution images of PC12 plasma membranes immunolabeled for syntaxin 1 before and after the addition of 150 μm Ca^2+^ (Fig. 1*A*). After analyzing at least 10 cell sheets from three different experiments, we observed that the average cluster density of syntaxin 1 increased from 3.3 ± 0.3 clusters μm^−2^ to 4.1 ± 0.4 clusters μm^−2^ upon calcium addition (Fig. 1*B*). In addition to this increase in density, Ca^2+^ increased the size of the domains from an average diameter of ∼90 nm in the absence of Ca^2+^ to ∼105 nm after Ca^2+^ addition (FWHM, Fig. 1*C*). Assuming circular domains, this equates to an ∼40% increase in domain area upon calcium addition, although this increase is an underestimate because domain sizes are overestimated by ∼5–10 nm because of the antibody staining. At our STED resolution, the sizes of the antibodies will contribute substantially to the measured domain sizes (the so-called “umbrella effect”) (7).

**FIGURE 2.**
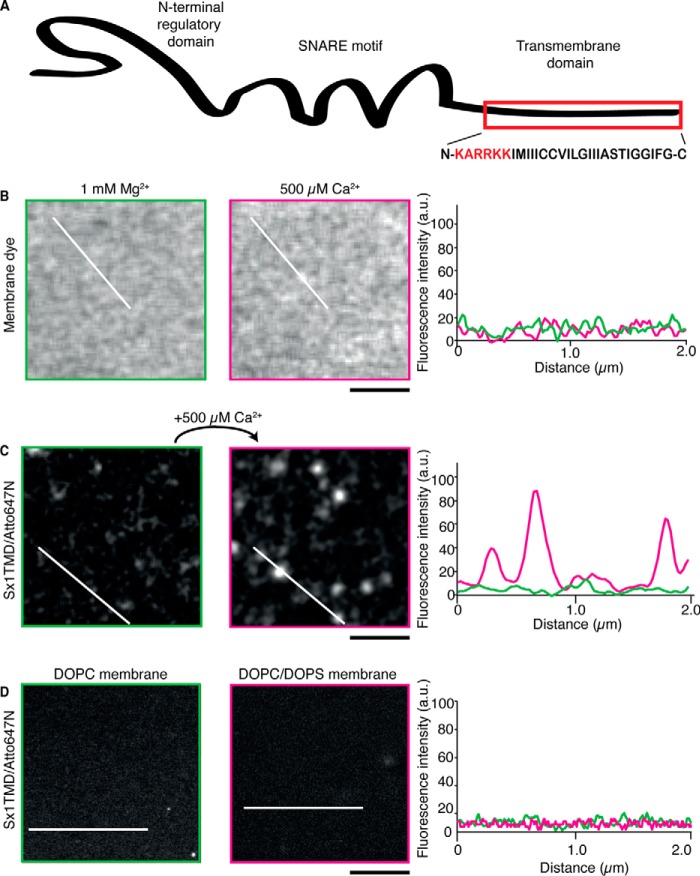
**Calcium-promoted syntaxin clustering requires PI(4,5)P_2_.** *A*, domain structure of syntaxin 1. The polybasic stretch juxtaposed to the transmembrane domain is marked in *red. B*, two-color STED images of glass-supported membranes containing sx-1 TMD (1:10,000 protein:lipid ratio) and composed of 97 mol% DOPC and 3 mol% PI(4,5)P_2_. Ceramide-Atto594 was used as a lipid marker (membrane dye). Buffers contained 1 mm Mg^2+^ (*left panel*, *green* in the intensity profile) or 500 μm Ca^2+^ (*center panel*, *magenta* in the intensity profile). Fluorescence intensity profiles along the cross-sections are indicated in the *right panel* in absolute units. *C*, the same as *B* but with sx-1 TMD labeled with Atto647N. *D*, the same as *B* but with membranes composed of pure DOPC (*left panel*) or 80 mol% DOPC and 20 mol% 1,2-dioleoyl-*sn-*glycero-3-phosphatidylserine (*DOPS*, *center panel*), both in the presence of 500 μm Ca^2+^. Representative data from multiple independent experiments are shown. Fluorescence intensity profiles along the cross-sections are indicated in the *right panel* in counts. *Scale bar* = 1 μm.

We then delineated the role of calcium on syntaxin 1 clustering in precisely controllable model membranes. We used fluorescently labeled syntaxin 1 TMD peptide (sx-1 TMD, residues 257–288). Apart from the polybasic linker region, this peptide does not contain the cytosolic domains (Fig. 2*A*), which allowed for exclusion of any contribution of cytosolic protein-protein interactions on syntaxin clustering. Our lipid mixtures for membrane reconstitutions contained DOPC without cholesterol. We recently showed that DOPC bilayers have a thickness of ∼3.5 nm, which matches the hydrophobic length of sx-1 TMD, and that this membrane system displays minimal clustering caused by hydrophobic mismatching (25). This model system therefore allowed us to focus on the effect of calcium on interactions between sx-1 TMD and PI(4,5)P_2_. In stacked lipid bilayers, the addition of Ca^2+^ did not affect the lipid bilayer structure as visualized by the fluorescently labeled lipid analogue ceramide-Atto590 (Fig. 2*B*). However, 500 μm of Ca^2+^ caused the clustering of reconstituted sx-1 TMD, provided PI(4,5)P_2_ was present in the membrane (Fig. 2*C*). We did not observe clustering of syntaxin in membranes composed of only DOPC or a mixture of 80 mol% DOPC and 20 mol% 1,2-dioleoyl-*sn-*glycero-3-phosphatidylserine (Fig. 2*D*).

**FIGURE 3.**
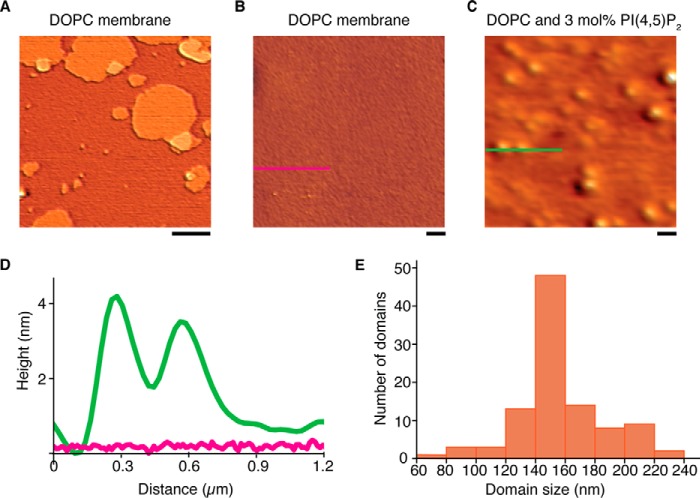
**Sx-1 TMD/PI(4,5)P_2_ domains reconstituted in lipid bilayers observed by AFM.** *A*, representative AFM image of stacked lipid bilayers on a glass support. Lipid bilayers were composed of pure DOPC and sx-1 TMD (1:10,000 protein:lipid ratio) in the presence of 500 μm Ca^2+^. Note that the membrane had patches of multiple layers on top of each other. *Scale bar* = 2 μm. *B*, magnification of the membrane region in *A. Scale bar* = 0.3 μm. *C*, the same as *B* but with bilayers containing 3 mol% PI(4,5)P_2_. *D*, height profiles along the cross-sections indicated in *B* (*pink*) and *C* (*green*). *E*, size distribution of sx-1 TMD domains from PI(4,5)P_2_-containing membranes (FWHM). Data are pooled from at least 10 independent reconstitutions.

To test whether the observed clusters were representing lateral membrane domains or small membrane vesicles on top of the bilayer, we used AFM. We prepared supported lipid bilayers with sx-1 TMD on plasma-treated glass surfaces (Fig. 3*A*). When the membrane was composed of only DOPC, scanning showed a homogenously flat surface regardless of the presence of calcium (Fig. 3, *B* and *D*). In contrast, and comparable with our fluorescence data, clusters were clearly observed when the membranes contained 3 mol% PI(4,5)P_2_, provided calcium was present (Fig. 3, *C* and *D*). Here the circular sx-1 TMD domains had an average size of 157 ± 2.7 nm (full width at half-maximum height, Fig. 3*E*), which is similar to the size determined by STED microscopy. In the absence of calcium, we did not observe clustering of syntaxin 1, regardless of the presence or absence of PI(4,5)P_2_. The average height of the domains was only 4 nm. Although this height might be taller than the expected dimensions of sx-1 TMD alone (∼2 nm), possibly because of buckling of the membrane, this height is far too small for any membrane vesicle. These experiments demonstrate that calcium can cluster sx-1 TMD in the presence of PI(4,5)P_2_ and supports our hypothesis.

**FIGURE 4.**
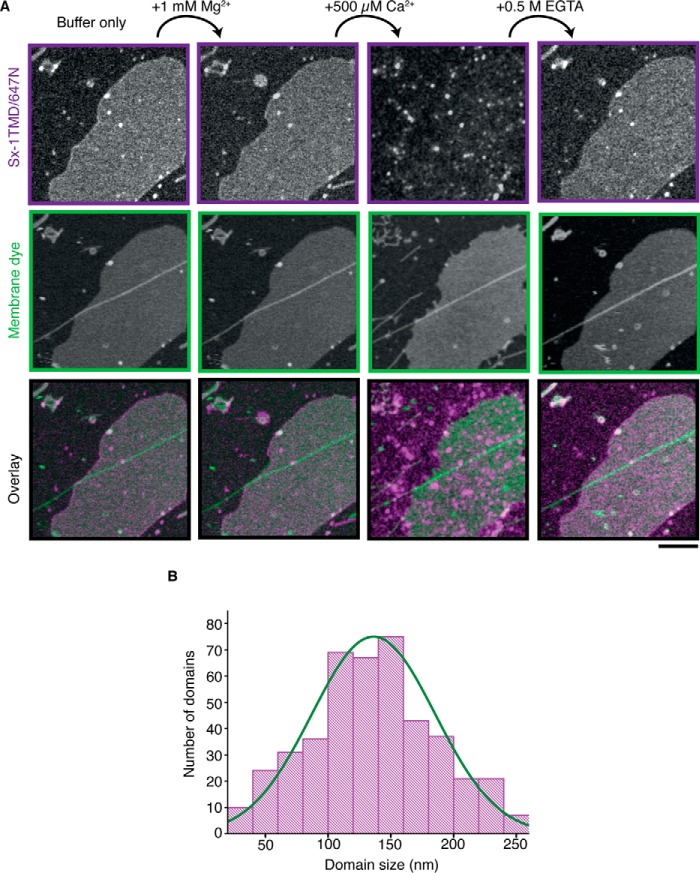
**Reversible clustering of syntaxin 1/PI(4,5)P_2_ domains induced by Ca^2+^.** *A*, representative series of STED images of membranes composed of 97 mol% DOPC and 3 mol% PI(4,5)P_2_ with sx-1 TMD reconstituted (protein:lipid ratio of 1:10,000, *magenta* in the overlay) and ceramide labeled with Atto590 (*green*, membrane dye). Mg^2+^ (1 mm), Ca^2+^ (500 μm), and EGTA (0.5 m) were added sequentially, and the same membrane area was imaged after each addition. *Scale bar* = 4 μm. *B*, size distribution of sx-1 TMD domains in the presence of 500 μm Ca^2+^ (FWHM, data from three independent reconstitutions).

**FIGURE 5.**
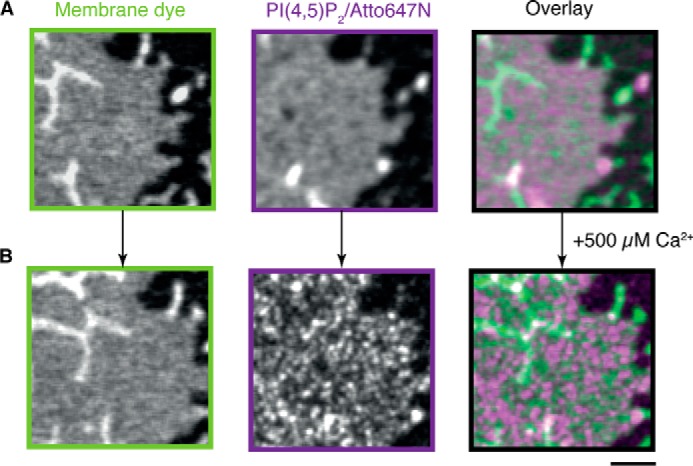
**Ca^2+^ induces PI(4,5)P_2_ clustering independently of sx-1 TMD.** STED images of membrane composed of 97 mol% DOPC, 3 mol% unlabeled PI(4,5)P_2_, 0.1 mol% PI(4,5)P_2_ labeled with Atto647N (*magenta* in the overlay), and 0.1 mol% DOPE-OG (Oregon green labeled 1,2-dioleoyl-*sn*-glycero-3-phosphatidylethanolamine; *green*, membrane dye). *A* and *B*, the same membrane area incubated in the absence and presence of 500 μm Ca^2+^, respectively. Representative images from multiple independent experiments are shown. *Scale bar* = 1 μm.

Next we tested the specificity and reversibility of calcium-triggered sx-1 TMD domain formation. To this end, we reconstituted sx-1 TMD in membranes that contained 3 mol% PI(4,5)P_2_ and recorded a series of STED images of the same membrane regions while changing the components of the buffer (Fig. 4). The addition of Mg^2+^ at a final concentration of 1 mm did not cause any clustering of the sx-1 TMD. However, the addition of 500 μm Ca^2+^ immediately triggered the formation of sx-1 TMD clusters with sizes between 70 and 200 nm (Fig. 4*B*). These domains were dependent on the presence of Ca^2+^ ions because chelating calcium with 0.5 m EGTA fully reversed sx-1 TMD clustering. The addition of 500 μm Ca^2+^ also triggered clustering of a PI(4,5)P_2_ variant containing an acyl chain labeled with Atto647N (Fig. 5), demonstrating that Ca^2+^ induced clustering not only of sx-1 TMD but also of PI(4,5)P_2_.

**FIGURE 6.**
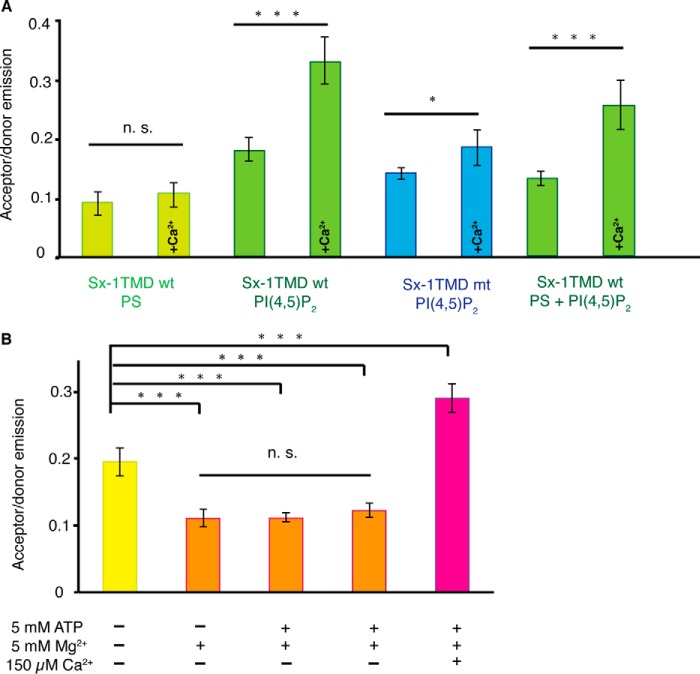
**Calcium induces coalescence of multiple syntaxin 1/PI(4,5)_2_ complexes.** *A*, membrane clustering by FRET on LUVs containing sx-1 TMD labeled with Rhodamine Red (donor fluorophore) and Atto647N (acceptor fluorophore). 150 μm Ca^2+^ significantly increased the FRET efficiency in the presence of 3 mol% PI(4,5)P_2_ but not in LUVs that contained 20 mol% 1,2-dioleoyl-*sn-*glycero-3-phosphatidylserine in the absence of PI(4,5)P_2_. Mutation of the sx-1 TMD (K264A, K265A; *Sx-1 TMD mt*) significantly reduced oligomerization both in the presence and absence of Ca^2+^ compared with the wild-type (*wt*). *B*, charge screening by ATP and Mg^2+^. LUVs were prepared as in *A*, and FRET was measured in buffer that was supplemented with 5 mm Mg^2+^, 5 mm ATP (disodium salt), or both and in the absence or presence of 150 μm Ca^2+^. *Error bars* indicate the range from three independent reconstitutions. ***, *p* < 0.001; *, *p* < 0.05%; *n.s.*, not significant; two-sided, paired *t* test).

To further characterize the molecular interactions between sx-1 TMD and the polar headgroup of PI(4,5)P_2_, we employed a recently developed FRET-based assay (15, 25). We reconstituted sx-1 TMD in LUVs with half of the peptide labeled with Rhodamine Red (FRET donor fluorophore) and the other half with Atto647N (acceptor fluorophore), both conjugated to the N-terminal end of the peptide. We measured the emission spectra in samples before and after the addition of 150 μm Ca^2+^. As expected, the polybasic juxtamembrane stretch of sx-1 TMD interacted with PI(4,5)P_2_, resulting in protein clustering, and this interaction was significantly increased after the addition of Ca^2+^ (Fig. 6*A*). To confirm the specific interaction of the polybasic juxtamembrane stretch of sx-1 TMD with PI(4,5)P_2_, we mutated two lysine residues located in the polybasic stretch to neutral alanines (K264A and K266A). Mutating these two lysines disrupts the interaction of syntaxin 1 with PI(4,5)P_2_ (15, 16). Indeed, this sx-1 TMD mutant showed reduced clustering with PI(4,5)P_2_, and the Ca^2+^ effect was also diminished. Sx-1 TMD clustering was also reduced in the absence of polyvalent PI(4,5)P_2_, and this clustering could not be rescued by monovalent phosphatidylserine (one negative charge, Fig. 6*A*).

In the final set of experiments, we investigated whether polyvalent ions other than calcium can decrease the electrostatic interactions responsible for syntaxin clustering by charge screening. To this end, we included both Mg^2+^ and ATP, which are present in the cytoplasm, at relatively high concentrations of 0.5–5 mm and 1–2 mm, respectively (30, 31). Indeed, in the absence of Ca^2+^, decreased clustering of sx-1 TMD was observed when 5 mm Mg^2+^ and/or 5 mm ATP was included in the buffer (Fig. 6*B*). However, Ca^2+^ was able to overcome this charge screening effect and increased sx-1 TMD clustering. Taken together, we conclude that Ca^2+^ can act as a charge bridge that merges multiple small sx-1 TMD/PI(4,5)P_2_ clusters into larger membrane domains.

## Discussion

In this study, we demonstrate that Ca^2+^ induces the coalescence of syntaxin-1/PI(4,5)P_2_ clusters into larger mesoscale domains. Three main conclusions can be drown from our findings. First, calcium only promotes clustering of the sx-1TMD construct in the presence of PI(4,5)P_2_. This corroborates our previous findings showing that PI(4,5)P_2_ is essential for clustering of syntaxin 1 and that targeting of the phosphatase domain of synaptojanin 1 (a PI(4,5)P_2_ phosphatase) to the plasma membrane causes the dispersion of syntaxin 1 clusters in the plasma membrane of PC12 cells (16).

Second, only Ca^2+^ but not Mg^2+^ promotes membrane clustering of syntaxin 1/PI(4,5)P_2_. This finding correlates well with several studies showing that Ca^2+^ specifically induces PI(4,5)P_2_ domains (19–23). As explained in these studies, the Ca^2+^ specificity is due to the charge density distributions and the matching of chelating properties between Ca^2+^ and the polynegative headgroup of PI(4,5)P_2_. Although in mammalian cells PI(4,5)P_2_ is the dominant phosphoinositide species in the plasma membrane (32) and is present at high concentrations in syntaxin 1 clusters (16), we do not expect that Ca^2+^-promoted clustering of syntaxin 1 is specific for PI(4,5)P_2_. Not only can calcium cluster other polyphosphoinositide species as well (19), but in fruit fly, syntaxin clustering at synaptic boutons is governed by phosphatidylinositol 3,4,5-triphosphate (17). Accordingly, not only syntaxin 1, but also other SNAREs, can interact with PI(4,5)P_2_. For instance, syntaxin 4 and the R-SNARE synaptobrevin 2 contain polybasic stretches adjacent to their TMDs that were shown to bind to PI(4,5)P_2_ (25, 33). Removal of these positive residues in synaptobrevin 2 inhibits exocytosis (33). It remains to be established whether synaptobrevin 2 can also be driven into clusters and, if so, whether such clustering is confined to the synaptobrevin pool in the plasma membrane or whether it also occurs in the membrane of secretory vesicles that are devoid of PI(4,5)P_2_.

**FIGURE 7.**
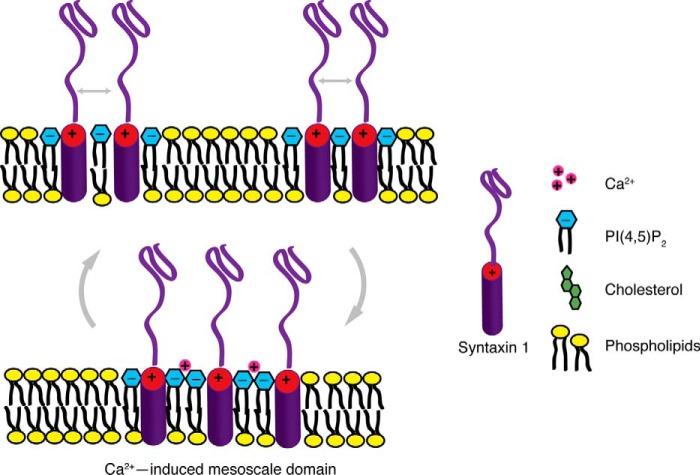
**Model of calcium-induced syntaxin domain formation.** Ca^2+^ specifically bridges PI(4,5)P_2_ molecules and induces the coalescence of syntaxin-1/PI(4,5)P_2_ clusters into larger mesoscale domains.

Third, the electrostatic interaction between the polybasic juxtamembrane stretch of syntaxin and the polynegative headgroup of PI(4,5)P_2_ are reduced by other polyvalent ions such as Mg^2+^ and ATP. The fact that Ca^2+^, at a much lower concentration, can overcome this electrostatic shielding shows that clustering is driven not only by bulk electrostatics but specifically involves a defined recognition of the participating molecules (Fig. 7). In PC12 cells, Ca^2+^ promotes clustering not only of syntaxin 1 but also of other SNAREs and integral and peripheral membrane proteins (18). Because many proteins directly or indirectly interact with PI(4,5)P_2_, including proteins that organize the cortical cytoskeleton (reviewed in Ref. 32, 34), Ca^2+^-induced PI(4,5)P_2_ clustering may well explain these structural rearrangements.

In this study, we observed increased clustering of syntaxin 1 at calcium concentrations of ∼500 μm in the microscopy-based experiments. In the more sensitive FRET assay using LUVs, clustering was already observed at lower calcium concentrations of ∼150 μm. These concentrations are higher than the global calcium concentration in synaptic boutons, which increases from less than 0.2 μm under resting conditions to ∼30 μm after membrane depolarization (35). However, much higher local calcium concentrations (several hundred micromolars) can be reached in the vicinity of synaptic calcium channels upon depolarization (36, 37). Syntaxin 1 domains are located in close proximity to calcium channels (38, 39), and these channels even physically interact with SNAREs (36), suggesting that Ca^2+^-dependent clustering does occur under physiological conditions.

What may be the functional significance of Ca^2+^-induced clustering of syntaxin? Ca^2+^/PI(4,5)P_2_-dependent clustering of syntaxin may play a role in exocytosis. We have recently shown that syntaxin 1/PI(4,5)P_2_ domains represent the preferred binding sites for the exocytotic calcium sensor synaptotagmin 1 (40, 41). This raises the possibility that a Ca^2+^-induced local increase in syntaxin density promotes SNARE complex formation at the site of release. Alternatively, syntaxin clustering by Ca^2+^ may remove excess SNAREs from the fusion site, thereby preventing hindrance of exocytosis by molecular crowding (5). A third possibility may be that clustering facilitates endocytosis. Ca^2+^-induced mesodomains may help to segregate plasma membrane proteins from the membrane proteins that are destined for endocytosis (42), thereby contributing to the rapid and high-fidelity recycling of synaptic vesicles. The main conclusion from this study is that ionic surface interactions between cations and polyanionic membrane lipids refine the lateral organization of the plasma membrane proteins, and this likely has implications for intracellular membrane trafficking.

## Author Contributions

D. M., A. H., G. v. d. B., and R. J. conceived and designed the experiments. M. P. and I. A. T. S. performed the AFM measurements. M. J. and U. D. synthesized the peptides. D. M. performed all other experiments and analyzed the data. D. M., G. v. d. B., and R. J. wrote the paper. All authors reviewed the results and approved the final version of the manuscript.

## Footnotes

3 The abbreviations used are:

PI(4,5)P_2_: phosphatidylinositol 4,5-bisphosphate
STED: stimulated emission depletion
AFM: atomic force microscopy
Fmoc: *N*-(9-fluorenyl)methoxycarbonyl
DOPC: 1,2-dioleoyl-*sn-*glycero-3-phosphatidylcholine
LUV: large unilamellar vesicle
FWHM: full width at half-maximum intensity
TMD: transmembrane domain.
